# Association between time-to-treatment and outcomes in non-small cell lung cancer: a systematic review

**DOI:** 10.1136/thoraxjnl-2021-216865

**Published:** 2021-08-17

**Authors:** Helen Hall, Adam Tocock, Sarah Burdett, David Fisher, William M Ricketts, John Robson, Thomas Round, Sarita Gorolay, Emma MacArthur, Donna Chung, Sam M Janes, Michael D Peake, Neal Navani

**Affiliations:** 1 Lungs for Living Research Centre, UCL Respiratory, UCL, London, UK; 2 Barts Health Knowledge and Library Services, Barts Health NHS Trust, London, UK; 3 MRC Clinical Trials Unit at UCL, UCL, London, UK; 4 Department of Respiratory Medicine, Barts Health NHS Trust, London, UK; 5 Centre for Primary Care and Public Health, Queen Mary University of London, London, UK; 6 School of Population Health and Environmental Sciences, King's College London, London, UK; 7 XX Place Health Centre, London Borough of Tower Hamlets, London, UK; 8 Centre for Cancer Outcomes, North Central and North East London Cancer Alliances, University College London Hospitals NHS Foundation Trust, London, UK; 9 Department of Respiratory Medicine, University of Leicester, Leicester, UK; 10 Department of Thoracic Medicine, University College London Hospital, London, UK

**Keywords:** non-small cell lung cancer

## Abstract

**Background:**

National targets for timely diagnosis and management of a potential cancer are driven in part by the perceived risk of disease progression during avoidable delays. However, it is unclear to what extent time-to-treatment impacts prognosis for patients with non-small cell lung cancer, with previous reviews reporting mixed or apparently paradoxical associations. This systematic review focuses on potential confounders in order to identify particular patient groups which may benefit most from timely delivery of care.

**Methods:**

Medline, EMBASE and Cochrane databases were searched for publications between January 2012 and October 2020, correlating timeliness in secondary care pathways to patient outcomes. The protocol is registered with PROSPERO (the International Prospective Register of Systematic Reviews; ID 99239). Prespecified factors (demographics, performance status, histology, stage and treatment) are examined through narrative synthesis.

**Results:**

Thirty-seven articles were included. All but two were observational. Timely care was generally associated with a worse prognosis in those with advanced stage disease (6/8 studies) but with better outcomes for patients with early-stage disease treated surgically (9/12 studies). In one study, patients with squamous cell carcinoma referred for stereotactic ablative radiotherapy benefited more from timely care, compared with patients with adenocarcinoma. One randomised controlled trial supported timeliness as being advantageous in those with stage I–IIIA disease.

**Conclusion:**

There are limitations to the available evidence, but observed trends suggest timeliness to be of particular importance in surgical candidates. In more advanced disease, survival trends are likely outweighed by symptom burden, performance status or clinical urgency dictating timeliness of treatment.

**Key messages**

**What is the key question?**

- To what extent does the timeliness of secondary care pathways impact outcomes in patients with non-small cell lung cancer.

**What is the bottom line?**

- Shorter times to treatment appear to be of greatest importance in those undergoing surgery treatment with curative intent, but do not appear to confer an advantage in patients with advanced disease.

**Why read on?**

- Our review is the first to address the evidence base for timeliness with a priori consideration for factors including demographics, histology, stage and treatment, and identifies patient groups at highest risk of adverse outcomes as a consequence of delays to treatment.

## Introduction

Lung cancer remains the most common cause of cancer-related death worldwide,^1^ largely due to the majority of patients being diagnosed with advanced stage disease, precluding treatment with curative intent.^2^ Instigating treatment as early as possible can maximise the benefits from curative intervention^3^ and, where advanced disease is already present, help initiate systemic therapies before clinical decline.^4^ In striving for this, primary care awareness and early referral,^5^ low-dose CT (LDCT) screening for high-risk groups^6 7^ and timeliness of secondary care pathways all require consideration.

Targets for timely investigation and management are driven in part by the risk of disease progression during avoidable delays. However, non-small cell lung cancer (NSCLC) displays both clinical and biological heterogeneity^8^ and some patients may benefit disproportionately from expedient care. Four previous reviews^9–12^ have explored the prognostic impact of timeliness in secondary care on patients with NSCLC. All report common limitations with heterogeneous evidence precluding quantitative analyses, and overall conclusions describe contradictory or paradoxical results with timeliness often associated with worse outcomes.^9–12^ A common emerging theme is the so-called ‘waiting-time paradox’,^12^ whereby more unwell patients with advanced disease receive more expedient treatment, thus suggesting a protective effect from treatment delays.^12 13^ Disentangling this requires consideration of factors likely to impact both time-to-treatment and clinical outcomes independently, but we are not aware of any previous reviews which have taken such an approach.

This systematic review aims to provide an updated overview of the literature, representative of current lung cancer management, and to identify patient groups most likely to benefit from expedient care. Focussing on secondary care pathways, we examine factors which may predict the greatest need for rapid investigation and treatment, the size of their impact on outcomes and how best to structure lung cancer services in order to optimise delivery of care.

## Methods

The protocol for this review was registered prospectively and is available online through PROSPERO (International Prospective Register of Systematic Reviews; ID 99239). Reporting standards are in accordance with Preferred Reporting Items for Systematic Reviews and Meta-Analyses (PRISMA) guidelines,^14^ with methodology and interpretation based on existing frameworks for narrative synthesis conduct.^15 16^

### Search strategy

Search strategies were devised for Medline, EMBASE and Cochrane with initial searches performed in July 2018 (online supplemental table 1A–C). Reference lists for included studies and previous reviews were hand-searched for additional relevant studies. Online registries (www.ClinicalTrials.gov and www.isrctn.com) were searched for works unpublished or in-progress. Searches were repeated on 6 October 2020 to capture interval publications.

10.1136/thoraxjnl-2021-216865.supp1 Supplementary data

Full inclusion and exclusion criteria are listed in table 1. Time intervals of interest included any from primary care referral to first treatment receipt, encompassing the ‘secondary care’ intervals as defined in the Aarhus statement.^17^ Studies published prior to January 2012 were excluded in order to minimise differences between reported data and current clinical practice, including routine use of positron emission tomography for staging, introduction of targeted therapies^18 19^ and staging from the International Association of Lung Cancer seventh^20^ or eighth edition.^21^

**Table 1:** PICOS question and inclusion/exclusion criteria

	Inclusion	Exclusion
*Patient*	Any patient aged ≥18 years Diagnosed with NSCLC Investigations and treatment performed in an elective secondary or tertiary care setting	NSCLC not examined in isolation from other cancer diagnoses Non-standard or emergency care pathways included Time intervals not measurable or not relevant to secondary/tertiary care
*Intervention*	Any with the intention of reducing part or all of time intervals from primary care referral to treatment	NA
*Control*	Usual care	NA
*Outcome*	Lung cancer-specific survival DFS OS Disease progression (eg, upstaging, change in proposed treatment)	Outcomes not directly correlated to timeliness
*Studies*	Any interventional or observational study Published January 2012–present	Not available in English language Abstract only No original data reported

DFS, disease-free survival; NSCLC, non-small cell lung cancer; OS, overall survival.

### Analysis

Themes for subgroup analysis were agreed a priori between the authors HH and NN, including demographics (age, gender, ethnicity and socioeconomic factors), clinical factors (comorbidities, symptoms and performance status), histological subtype, stage and treatment modality. Data were tabulated from all included studies to include: population, sample size, study design and data source, measured time intervals, definitions of ‘delay’ and outcome measures (online supplemental table 2).

Following abstraction, studies were categorised according to relevance to the above themes. Methodological parameters including reported time intervals, definition of delays and reported outcome measures were compared for any studies reporting data relevant to a specific subgroup, but heterogeneity between studies precluded quantitative analyses. Overall findings are explored in a narrative synthesis with trends summarised via vote counting according to direction of effect.^15^ Analyses were defined under the following terms:

- ‘Timely care’ or ‘timeliness’ described any aspect of care delivered within a time interval which was shorter than that experienced by a comparatively ‘delayed’ group, including differences in median time intervals or time intervals falling within a predefined threshold (eg, within a defined number of weeks or a guideline-defined target).
- ‘Timeliness advantageous’—faster measured time intervals associated with improved outcomes
- ‘Timeliness deleterious’—faster measured time intervals associated with worse outcomes
- ‘Mixed’—trends of varying direction of effect reported within different subgroups of one study
- ‘Non-significant’—no statistically significant trends reported

Study outcomes are described under the above terms for observed trends as per the primary outcome of the study. Where different subgroups of interest are explored within the narrative synthesis, the reported trends reflect the observed association within that subgroup only.

### Bias assessment

Studies were assessed for potential sources of bias, including completeness and clarity of data sources and methodology, representativeness of the target population, management of missing data, defined time intervals and consideration of confounding factors including potential ‘waiting time bias’ (online supplemental table 5A-B). Bias assessment criteria were derived from the Strengthening the Reporting of Observational Studies in Epidemiology^22^ and Aarhus statements,^17^ and previous similarly structured reviews.^12 23^ For interventional trials, the 2011 Cochrane ‘Risk of bias’ tool was used.^24^

### Study selection and characteristics

Literature search outcomes are summarised in online supplemental figure 1. Searches for unpublished works found two further trials, one withdrawn (ClinicalTrials.gov: NCT1946490) and the second currently recruiting (NCT03535766). Thirty-seven papers met the criteria for inclusion, of which all but five^25–29^ included findings relevant to a subgroup of interest. One paper could not be obtained for review of the full manuscript.^30^

## Results

We report an overview of the included studies, with subsequent exploration of predefined themes: demographics, clinical factors, histology and stage/treatment. Two interventional papers are then considered separately: one randomised controlled trial (RCT) and one ‘quasi-experimental’ case–control study.

### Overview of included studies

All but two of the included papers are observational. Ten report data from Europe (four from the UK), 19 from North America, 5 from Asia, 1 from Australasia and 2 from South America. Sixteen were single-centre studies, 4 were multicentre studies and 16 report registry data. One reports both an analysis of registry data and a single-centre cohort study.^31^

Timeliness measures are variably defined as dichotomous (15 papers), categorical (8 papers), continuous (19 papers) or guideline concordant versus non-concordant (5 papers). Twenty-four papers include measures of the defined time intervals (online supplemental figure 2). Thirty studies report survival as an outcome measure, 10 report upstaging and 3 report change in treatment intent. Overall, timely care was reported as advantageous in 13 papers, deleterious in 9 and non-significant or mixed in 15 (online supplemental table 2).

### Demographics

Five studies focus on demographic factors in their primary analyses.^32–37^ Di Girolamo *et al* report all age groups to experience worse survival with receipt of guideline-concordant care compared with those receiving delayed treatment.^32^ Three other papers report data on patients aged >66 years only with varied conclusions. Nadpara *et al* examines trends in both regional and national registry datasets, concluding timely care to be independently associated with worse survival in the former^34^ but finding no significant association in the latter.^33^ Gomez *et al* similarly included only participants aged >66, concluding timeliness to be advantageous in early-stage disease but more equivocal in regional and advanced disease.^36^

Forrest *et al* 35 examine the impact of socioeconomic position (SEP), concluding lower SEP groups to be independently associated with worse survival; however, the authors attribute this to inequalities in performance status and treatment type rather than receipt of timely care. Napolitano *et al* explore the impact of private versus Medicare insurance in a US single-centre cohort (n=112), reporting faster times from diagnostic CT to surgery in those with private insurance (66 vs 86 days, p=0.03); however although there was a trend towards fewer privately insured patients being upstaged, this did not meet statistical significance (22.9% vs 31.8%, p=0.32).^37^

A further nine papers include multivariable analyses controlling for factors including age,^27 31 35 38–42^ gender,^27 38–40 42^ ethnicity,^31 39 40 42^ income,^31 38 40^ deprivation index^42^ and education,^40^ but adjusting for these factors did not influence the reported associations between timeliness and outcomes.

### Clinical features and comorbidities

Only one study addresses symptomatology at presentation.^42 43^ Redaniel *et al* 42 examine the impact of ‘alert’ clinical features (haemoptysis, stridor or superior vena cava obstruction), observing an independent association between improved survival and longer time to diagnosis only in those without such symptoms. Several other studies report outcomes in multivariable analyses controlling for clinical factors including comorbidity scores^27 31 35 36 41 42^ and performance status.^27 44^ Of these, the only significant association is reported by Radzikowska *et al*, who find timeliness associated with worse survival only in patients with performance status of 2 (HR: 1.28, p<0.001).^44^

### Histology

Seven papers control for histology in multivariable analysis, but none report this to be a significant factor.^27 28 35 39 42 45 46^ Only Murai *et al*’s study of patients referred for stereotactic ablative radiotherapy (SABR) reports a significant association, with higher rates of upstaging seen in those with squamous cell differentiation (29%) versus adenocarcinoma (5%) in patients waiting longest.^47^

### Stage

Twenty-six papers stratify outcomes by disease stage (table 2). In addition, four papers report multivariable analyses controlling for stage among other factors, and found no significant impact.^27 34 35 42^

**Table 2:** Summary of evidence by stage–

	Timeliness advantageous	Non-significant	Timeliness deleterious	Mixed
Localised disease				
All treatment	Murai *et al* 2012^47^	Nadpara *et al* 2015 (I)^33^	Vinod *et al* 2017 (palliative only)^53^	**Di Girolamo *et al* 2018 (I)** 32
	Wang *et al* 2012 (I–III)^48^	Bullard *et al* 2017 (I)^39^	**Di Girolamo *et al* 2018 (II)** 32	
	**Gomez *et al* 2015 ('Localised')** 36	Frelinghuysen *et al* 2017^41^		
	Navani 2015 (I–IIIA)^52^	Vinod *et al* 2017 (I–II)^53^		
	Kasymjanova *et al* 2017 (I–IIB)^49^	Abrao *et al* 2018 (I)^46^		
	Abrao *et al* 2018 (II)^46^	Ha *et al* 2018 (I–IIIA)^54^		
	Khorana *et al* 2019 (I–II)^40^ *			
	**Cushman *et al* 2020 (I–II**)^50^ *			
	**Tsai *et al* 2020 (I–II**)^51^			
Surgery only	**Yun *et al* 2012** 59	Coughlin *et al* 2015 (I)^45^		
	Kanarek *et al* 2014 (I–IIIA)^66^	Samson *et al* 2015 (single centre)^31^		
	**Bott *et al* 2015 (I**)^58^ *	Shin *et al* 2013 ('Local’)^38^		
	Coughlin *et al* 2015 (II)^45^	Navani *et al* 2015 (I–IIIA)^52^		
	**Samson *et al* 2015 (registry)** 31 *	Vinod *et al* 2017^53^		
	**Yang *et al* 2017 (IA**)^67^ *			
	**Khorana *et al* 2019 (I+II**)^40^ *			
	Huang *et al* 2020 (stage I)^62^			
	**Cushman *et al* 2020 (stage I–IIIA**)^50^ *			
Regional disease	Kasymjanova *et al* 2017^49^	**Gomez *et al* 2015** 36	Nadpara *et al* 2015^33^	Wai *et al* 2012^57^
	**Tsai *et al* 2020** 51	Robinson *et al* 2015^55^	Vinod *et al* 2017^53^	Di Girolamo *et al* 2018^32^
		Friedman *et al* 2016^56^		
		Bullard *et al* 2017^39^		
		Abrao *et al* 2018^46^		
Advanced disease	**Gomez *et al* 2015 (survival >1 year**)^36^	**Tsai *et al* 2020** 51	Nadpara *et al* 2015^33^	
			**Gomez *et al* 2015 (survival <1 year**)^36^	
			Kasymjanova *et al* 2017^49^	
			Vinod *et al* 2017^53^	
			Bullard *et al* 2017^39^	
			Abrao *et al* 2018^46^	
			Di Girolamo *et al* 2018^32^	

Bold denotes papers with n>1000.

Disease stage/subgroup in parenthesis.

*Papers reporting data from NCDB.

NCDB, National Cancer Database.

#### Localised disease

‘Localised’ disease outcomes are reported in 23 papers including three which group all stage I–IIIA treated with curative intent. Fourteen report outcomes without differentiation by treatment modality (online supplemental table 3A) including two studies reporting rates of upstaging in patients referred for SABR, but not the outcome of SABR delivery per se.^41 47^ Twelve studies report outcome data specific to patients undergoing surgery (online supplemental table 3D). Four studies include data for both all treatment modalities and surgical subgroups, and are therefore listed in both tables.

Where all treatment modalities in localised disease are included, the majority of studies find timeliness to be advantageous^36 40 47–52^ (including one RCT,^52^ discussed below), or do not meet statistical significance.^33 39 41 53 54^ Abrao *et al* find timeliness only to be advantageous in those with stage II disease.^46^ Only Di Girolamo *et al* demonstrated persistent association between timeliness and worse outcomes in stage I and II disease.^32^ Outcomes specific to surgery recipients are discussed below.

#### Regional disease

Twelve studies refer to either ‘regional’ or stage III disease in isolation, with more equivocal trends in observations (online supplemental table 3B). Two studies report timeliness to be advantageous,^49 51^ four find timeliness to be deleterious in one or more measured time interval,^32 33 50 53^ five find no significant association^36 39 46 55 56^ and one reports mixed trends across different measures of delay.^57^ Robinson *et al* find a significant proportion of patients experience clinical deterioration impacting their treatment intent, but wait times were no different to those with no significant deterioration.^55^ Wai *et al* find patients receiving radical chemoradiotherapy rather than palliative interventions experienced faster times from diagnosis to cancer centre referral, but longer intervals between oncology review and first treatment.^57^ However, in this paper a significant proportion of controls do not have data for performance status, purportedly a factor used for matching case to control.

#### Advanced disease

Outcomes in advanced disease (stage IV) are reported by eight studies, of which the only group seen to benefit from timely care are those described in the study by Gomez *et al* 36 as surviving >12 months from diagnosis (online supplemental table 3C). One paper reports no significant association,^51^ otherwise trends support a deleterious effect of timeliness, though only one paper controls for treatment modality.^53^

### Treatment

#### Surgery

Twelve papers report surgical outcomes, nine concluding timeliness to be advantageous, primarily large studies reporting registry data (online supplemental table 3D). Of note, five of these studies use registry data from National Cancer Database (online supplemental table 4), raising potential for individual patient data to be replicated between studies, particularly those of Samson *et al* and Bott *et al*.^31 58^

RCT evidence from Navani *et al* did not show statistical significance for the association between timeliness and survival in a subgroup of 29 patients treated surgically (HR: 0.37, 95% CI: 0.1 to 1.32).^52^ Two relatively small studies are similarly inconclusive^38 53^ and a third reports timeliness to only be of significance in patients with stage II disease (vs stage I).^45^ Yun *et al* report significantly increasing impact of surgical delays for those treated at low-volume surgical centres.^59^ Only one study found a potential increase in risk of upstaging with timeliness, however there was no associated increased risk of mortality in the same cohort.^31^

#### Systemic therapy and palliative care

Delays of >45 days from diagnosis to receipt of chemoradiotherapy were associated with improved survival versus timely treatment with HR 0.88 (0.83–0.93) in one study.^50^ Vinod *et al* note a statistically significant trend towards worse outcomes in those with stage I–III disease receiving palliative care faster, but did not find significant trends for any other treatment modality.^53^ No papers were found which report outcomes from targeted therapies or immunotherapy.

### Interventional trials

One RCT^52^ and one ‘quasi-experimental’ case–control study^60^ were identified. The multicentre Lung-BOOST trial^52^ randomised 133 patients (96 with latterly confirmed stage I–IIIA NSCLC) to endobronchial ultrasound-guided transbronchial needle aspiration (EBUS-TBNA) or conventional diagnosis and staging (CDS). Time to treatment decision in the EBUS-TBNA group was significantly faster that the CDS group (median 15 vs 30 days, p<0.0002). In a post-hoc analysis, longer median survival was observed (503 vs 312 days, p=0.038) in the EBUS-TBNA group versus CDS, though the authors suggest this may in part be attributable to increased pre-operative mediastinal staging resulting in a refined population undergoing surgery, conferring a survival benefit.

Selva *et al* 60 evaluated the impact of a ‘rapid diagnosis and treatment programme’ against usual care (control data taken from retrospective records). Although introduction of the pathway reduced the diagnosis-to-treatment interval by 9 days, in multivariate analysis this difference was not significant, and no significant difference in stage distribution was observed.

## Discussion

### Summary of evidence

The trends seen in these observational studies plus one RCT suggest timeliness is of importance in patients with lung cancer with early-stage disease, particularly those undergoing surgery. In advanced disease, the available evidence supports the previously described ‘waiting-time bias’, accounted for by both urgency of intervention in those who are most symptomatic and palliative interventions being typically delivered more rapidly than curative following confirmed diagnosis.^32^ Isolated studies suggest patients with performance status of 2 (57) or squamous cell cancer as compared with adenocarcinoma^59^ may benefit disproportionately from expedited care, but these findings are not observed consistently.^41^

Outcomes in early-stage disease are not consistent across the reviewed evidence. Di Girolamo *et al*’s 2018 review of UK cancer registry data reports the impact of receiving care within standard national targets,^61^ concluding a harmful impact of faster treatment across all stages of NSCLC in spite of excluding those who died within 90 days of diagnosis. One explanation offered is that treatments delivered fastest—palliative care, active monitoring or ‘patient refusal’—confer a worse prognosis. We note 17.6% of those with stage I disease did not receive any active treatment which may account for some degree of the observed association. Data as regards the outlier values within the longest treatment intervals are not presented by Di Girolamo *et al*, but a possibility is that those with indolent lesions who undergo substantial periods of surveillance between initial radiological ‘diagnosis’ and treatment may also skew the data to suggest that longer times to treatment improve outcomes as has been reported elsewhere.^62–64^

### Evidence quality and potential bias

Of the available evidence many studies are observational in design, and only one RCT is identified (online supplemental table 5A, B). Several studies rely on registry data which may be limited in terms of completeness and representativeness,^65^ furthermore time interval measures may be extrapolated from indirect sources (eg, dates of insurance claims for consultations). Equally, smaller studies may not be sufficiently powered to detect mortality signals. The reporting of delayed versus timely care is highly variable across the included studies, thus creating difficulty in establishing comparative trends (online supplemental table 2). It is worth noting that many studies report the impact of a binary definition of treatment defined a priori, given the approach taken towards quantifying delays can in itself lead to inconsistency in reported trends.^66^

Substantial efforts in this study have been made to ensure completeness of the literature review and multiple papers not included in previous systematic reviews have been identified. The review protocol, including research questions and thematic analyses, were devised a priori with the aim of minimising reporting bias during narrative synthesis. No issues were encountered as regards accessing studies potentially appropriate for inclusion, but we have not sought individual patient data from the authors of any included studies. We did not find a significant number of works in progress or withdrawn to suggest publication bias to be a significant issue. We note the degree of overlap between some large registry-based studies,^31 40 58 67^ which may bias the overall weight of evidence particularly in surgical recipients; however, the contributions taken by different groups in their approach to these data are informative in our subgroup analyses and therefore warrant inclusion.

### Generalisability

The presented data cover a broad spectrum of practice, both by geography, healthcare models and time, though there are some limitations to this. The available data are predominantly from North American and European populations, with lesser representation of South American and Asian data and no studies found reporting outcomes from African cohorts. However a number of studies report data controlling for ethnicity and none find this to influence associations with timeliness. Despite our described restrictions on publication date, some included studies report data from >20 years ago, encompassing a period of variation in clinical practice, staging iterations and treatment guidelines.^31 44 57^ The structure of the patient pathway from symptoms to treatment varies internationally and we recognise some of the described diagnostic pathways may not be applicable to all systems (eg, direct referral from primary care to thoracic surgery^37^). However, while these differences preclude meaningful quantitative analyses, the relatively consistent trends observed suggest our overall conclusions are likely to be valid across the majority of current healthcare settings.

Two key patient groups are not addressed: those receiving targeted therapies and immune checkpoint inhibitors and those diagnosed via LDCT screening pathways. Cancers diagnosed via LDCT screening programmes may be more indolent and therefore warrant separate consideration,^68^ but we found no studies which address timeliness in the management of such lesions in secondary care. Similarly, only two studies mention patients receiving targeted therapies, now widely recognised as standard of care in many patients with advanced disease.^49 53^ Timeliness may be key to reduce the risk of clinical deterioration precluding these treatments, but we have not found an evidence base to address this question. Equally, the additional time required for mutational analysis prior to patients receiving these therapies could also contribute to an apparently protective impact of longer diagnostic intervals if treatment modality is not controlled for.^53^

### Implications for practice and policy

Our observations from the available evidence suggest that patients referred for surgery may benefit most from shorter times to intervention. The available data are not consistent enough to recommend specific time intervals, but at worst a prognostic impact may be seen with delays of just 7 days from diagnosis to treatment^51^ with other studies suggest a cumulative impact of worse prognosis with every week’s delay from diagnosis to treatment.^40 66^

These findings suggest that the targets laid out in the National Optimal Lung Cancer Pathway, targeting a ‘referral-to-diagnosis’ interval of 28 days and ‘referral-to-treatment’ of 49 days,^69^ will give rise to a downstream improvement in NSCLC survival particularly for those with early-stage disease. The impact for those with advanced disease is less certain; our conclusions highlight the overwhelming impact of confounding factors on observed trends in this group, and further work is required to appreciate the role of timeliness as regards the risk of clinical deterioration and subsequent impact on emergency admissions or planned treatments.

For all stages of disease, other factors warrant consideration in determining targets for optimal delivery of care. Timely care may reduce anxiety and improve overall patient experiences for many, though equally may contribute to a sense of bewilderment and complexity for some.^70^ Equally, pressure to deliver surgery within a certain timeframe may limit opportunity for ‘prehabilitation’ and smoking cessation and thus impact resection rates and post-operative outcomes in high-risk patients.^71–73^

## Conclusion

Although there are inconsistencies and limitations to the available evidence, the observed trends support timeliness as being associated with better outcomes in patients with early-stage disease, particularly those undergoing surgery. In patients with advanced disease, the benefit of urgent intervention is likely to be outweighed by other clinical and biological factors. Currently, evidence is lacking as regards the role of timeliness for patients receiving targeted therapies or immunotherapy, or those diagnosed via lung cancer screening programmes. Rapid pathways to treatment should be implemented to improve outcomes for patients with early-stage lung cancer.

## Data Availability

All data relevant to the study are included in the article or uploaded as supplementary information.
