# Supplementary material for: Association between time-to-treatment and outcomes in non-small cell lung cancer: a systematic review
Source: Thorax. 2021 Aug 17;77(8):762–8. doi: 10.1136/thoraxjnl-2021-216865 (PMC9340041; doi:10.1136/thoraxjnl-2021-216865)
Supplement: Supplementary data [file thoraxjnl-2021-216865supp001.pdf]

Title: Association between time-to-treatment and outcomes in non-small cell lung cancer: a systematic review

Authors:

Helen K Hall, Adam Tocock, Sarah Burdett, David Fisher, William Ricketts, John Robson, Thomas Round, Sarita Gorolay, Emma MacArthur, Donna Chung, Sam M Janes, Michael D Peake, Neal Navani

Online Supplementary materials

Table 1a: Database search methodology (Medline)

Table 1b: Database search methodology (EMBASE)

Table 1c: Database search methodology (Cochrane)

Figure 1: PRISMA flowchart

Table 2: Summary and abstraction of included studies

Figure 2: Reported time intervals

Table 3a: Summary of evidence in early disease

Table 3b: Summary of evidence in regional disease

Table 3c: Summary of evidence in advanced disease

Table 3d: Summary of evidence in surgical cohorts

Table 4: Comparison of studies utilising National Cancer Database

Table 5a: Bias assessment for observational studies

Table 5b: Bias assessment for randomised controlled trials

Table 1a: Database search methodology – outcomes of first search (Medline)

1. ((lung* AND (carcinogen* OR sarcom* OR metasta* OR tumor* OR tumour* OR 2arcinoma* OR cancer* OR neoplasm*)) AND diagnos*).ti,ab 47802
2. Exp *"LUNG NEOPLASMS"/ AND exp *DIAGNOSIS/ 22558
3. Exp *"LUNG NEOPLASMS"/di 15129
4. (44 OR 45 OR 46) 72249
5. Exp *"TIME FACTORS"/ 2019
6. Exp *"TIME-TO-TREATMENT"/ 1557
7. (delay* OR timely OR timeliness OR speed*).ti,ab 616523
8. ((("2 week*" OR "two week*") ADJ wait*) OR 2ww OR tww).ti,ab 234
9. (48 OR 49 OR 50 OR 51) 619407
10. (47 AND 52) 1899
11. (outcome*).ti,ab 1392388
12. Exp "PATIENT OUTCOME ASSESSMENT"/ 5386
13. (70 OR 71) 1393537
14. (survival).ti,ab 802667
15. Exp MORTALITY/ 342122
16. (mortality).ti,ab 634887
17. (73 OR 74 OR 75) 1474956
18. (72 OR 76) 2540309
19. (53 AND 77) 696

Table 1b: Database search strategy – outcomes of first search (EMBASE)

1. ((lung* AND (carcinogen* OR sarcom* OR metasta* OR tumor* OR tumour* OR 2arcinoma* OR cancer* OR neoplasm*)) AND diagnos*).ti,ab 85332
2. Exp *"LUNG CANCER"/ AND exp *DIAGNOSIS/ 18020
3. Exp *"LUNG CANCER"/di 21226
4. (54 OR 55 OR 56) 106387
5. (delay* OR time* OR timeliness).ti 344301
6. ((("2 week*" OR "two week*") ADJ wait*) OR 2ww OR tww).ti,ab 565
7. Exp "TIME FACTOR"/ 19038
8. (58 OR 59 OR 60) 361215
9. (57 AND 61) 1409
10. (outcome*).ti,ab 2039908
11. Exp "TREATMENT OUTCOME"/ 1396119
12. (79 OR 80) 2806681
13. (survival).ti,ab 1167404
14. (mortality).ti,ab 922767
15. Exp SURVIVAL/ 941339
16. Exp MORTALITY/ 941184
17. (82 OR 83 OR 84 OR 85) 2379942
18. (81 OR 86) 4473764
19. (62 AND 87) 627

Hall H, *et al. Thorax* 2022; 77:762–768. doi: 10.1136/thoraxjnl-2021-216865

Table 1c: Database search strategy – outcomes of first search(Cochrane)

#1 (((lung* AND (carcinogen* OR sarcom* OR metasta* OR tumor* OR tumour* OR 3arcinoma* OR cancer* OR neoplasm*)) AND diagnos*)):ti,ab,kw 5094
#2 MeSH descriptor: [Lung Neoplasms] explode all trees 6733
#3 MeSH descriptor: [Diagnosis] explode all trees 312508
#4 #2 and #3 3251
#5 MeSH descriptor: [Lung Neoplasms] explode all trees and with qualifier(s): [diagnosis – DI] 275
#6 #1 or #4 or #5 7504
#7 MeSH descriptor: [Time Factors] explode all trees 62064
#8 MeSH descriptor: [Time-to-Treatment] explode all trees 237
#9 (delay* OR timely* OR timeliness OR speed*):ti,ab,kw 57111
#10 (((“2 week” or “2 weeks” OR “two week” or “two weeks”) and wait*) OR 2ww OR tww):ti,ab,kw 567
#11 #7 or #8 or #9 or #10 114955
#12 #6 and #11 650
#13 (outcome*):ti,ab,kw 496294
#14 MeSH descriptor: [Patient Outcome Assessment] explode all trees 553
#15 #13 or #14 496302
#16 survival or mortality 155298
#17 MeSH descriptor: [Survival] explode all trees 128
#18 #16 or #17 155298
#19 #15 or #17 496348
#20 #12 and #19 391
#21 #20 with Cochrane Library publication date from Jan 2012 to present 258

Hall H, *et al. Thorax* 2022; 77:762–768. doi: 10.1136/thoraxjnl-2021-216865

Figure 1: PRISMA flowchart

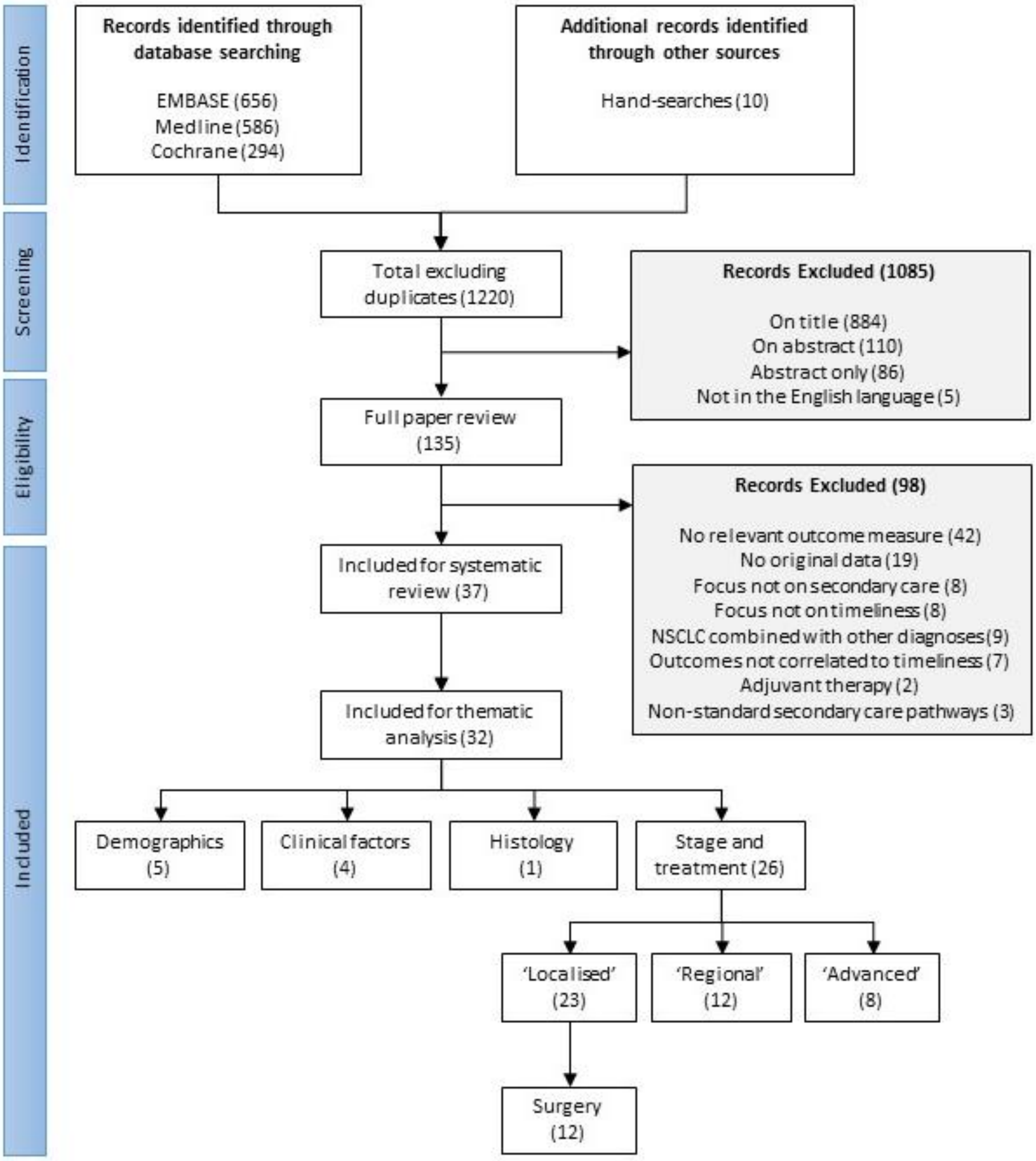

Table 2: Summary and abstraction of included studies

Reference	Population and NSCLC* sample size	Design and data source	Measured time intervals	Outcome measure	Trend (overall)	Results summary	Sub-group analysis
**Abrao 2017**(25)<br><br>Brazil	All LC, previously untreated<br><br>n=435	Single centre, observational cohort study<br><br>2008-2014	First review to diagnosis, diagnosis to treatment	LC-specific survival	Timeliness deleterious	Worse LC-specific survival seen in those with <1.5 months from diagnosis to first treatment in multivariate analysis (13 vs 4 months, p<0.01).	Nil
**Abrao 2018**(46)<br><br>Brazil	All NSCLC<br><br>n=359	Single centre, observational cohort study<br><br>2008 - 2014	Diagnosis to treatment	OS	Timeliness deleterious	Overall intervals of >2 months from diagnosis to treatment was protective, with adjusted HR 0.75 (p=0.001)	Stage (localised, regional, advanced)
**Bott 2015**(56)<br><br>USA	Clinical stage 1 NSCLC undergoing curative resection<br><br>n=55,653	Registry (NCDB)<br><br>1998 - 2010	Histological diagnosis to surgery	Pathological upstaging	Timeliness advantageous	A delay of >8 weeks from diagnosis to surgery was associated with higher risk of pathological upstaging (OR 1.10)	Stage (localised), surgery
**Brocken 2012**(26)<br><br>Netherlands	All consecutive referrals to a single centre lung MDT (indeterminate nodules excluded)<br><br>n=261	Single centre, observational cohort study<br><br>1999 - 2009	PC referral to first review; first review to diagnosis; PC referral to treatment; diagnosis to treatment	PFS, OS	Non-significant	Delays not associated with disease stage or survival	Nil
**Bullard 2017**(39)<br><br>USA	All NSCLC<br><br>n=746	Registry (South Carolina Central Cancer Registry)<br><br>2005-2010	Diagnosis to treatment	OS	Timeliness deleterious	Worse survival seen with diagnosis to treatment intervals of <6 weeks in advanced disease	Stage (localised, regional, advanced)
**Coughlin 2015**(45)<br><br>Canada	Clinical stage I-II NSCLC undergoing surgical resection<br><br>n=222	Single centre, observational cohort study<br><br>2010 - 2011	Treatment decision to treatment	Pathological upstaging	Timeliness advantageous	In stage 2 disease, delays of >8 weeks were associated with increased risk of pathological upstaging and worse survival. Did not meet significance in stage 1 disease.	Stage (localised), surgery
**Cushman 2020**(52)<br><br>USA	Histologically confirmed stage I-IIIB NSCLC treated with curative intent, excluding time to treatment >365 days<br><br>n=140,455	Registry (NCDB)<br><br>2004 - 2015	Diagnosis to treatment	OS	Timeliness advantageous	>45 days from diagnosis to treatment associated with median survival 61.5 months vs 70.2 for timely care (p < 0.001)	Stage (localised, regional), surgery

**Di Girolamo 2018**(67)<br><br>UK	All NSCLC<br><br>n=121,963	Registry (CWT, NCRAS)<br><br>2009 - 2013	PC referral to first review; diagnosis to treatment; PC referral to treatment	One-year net survival (adjusted for competing causes of mortality)	Timeliness deleterious	One-year survival worse in those treated within 31- and 62-day targets	Demographics, stage (localised, regional, advanced)
**Forrest 2015**(35)<br><br>UK	All lung cancer, any active treatment.<br><br>n=12,152	Registry (Lung Cancer Audit; Northern and Yorkshire Cancer Registry and Information Centre; Hospital Episode Statistics)<br><br>2006-2009	PC referral to first review; diagnosis to treatment; PC referral to treatment	OS	Timeliness deleterious	Treatment within 31 days of diagnosis was associated with worse 2-year survival (OR 0.37)	Demographics
**Frelinghuysen 2017**(41)<br><br>Netherlands	Inoperable NSCLC planned for SABR<br><br>n=123	Single centre, observational cohort study<br><br>2005 - 2008	Diagnostic CT to treatment planning CT (ISI)<br><br>Excl if ISI <25 days	Upstaging, OS	Non-significant	Risk of upstaging was not correlated to longer time to treatment	Stage (localised)
**Friedman 2016**(62)<br><br>USA	All stage III NSCLC<br><br>n=109	Single centre case:control, comparing referral to single clinician versus cancer board	First clinical review to treatment	OS	Non-significant	Patients seen by MTD experienced faster treatment with borderline significant improved median survival (14 vs 17 months, p = 0.054)	Stage (regional)
**Geiger 2014**(29)<br><br>USA	Non-metastatic NSCLC<br><br>n=47	Single centre, observational cohort study<br><br>2009 – 2011	Diagnostic CT to treatment planning CT (ISI)<br><br>Excl if ISI >120 days	Upstaging<br><br>Change in treatment plan	Non-significant	Upstaging observed in 21% of those with ISI <43 days vs 30% of those with ISI >43 days, p = not given	Nil
**Gomez 2015**(36)<br>USA	All NSCLC with Medicare claims<br><br>n=28,732	Registry (Medicare claims)<br><br>2004 - 2007	Diagnosis to treatment	OS	Mixed	Treatment within 35 days of diagnosis associated with improved survival in those with localised disease and those with advanced disease who survived >1 year (HR 0.86 for both groups) but worse in those with advanced disease surviving <1 year (HR 1.35)	Demographics, stage (localised, regional, advanced)
**Gonzalez-Barcala 2014**(27)<br><br>Spain	Pathologically confirmed LC<br><br>n=262	Single centre, observational cohort study<br><br>2005-2008	First review to diagnosis, diagnosis to treatment	Survival NOS	Timeliness deleterious	Survival is improved in patients waiting >61 days from diagnosis to treatment, but time from first review to diagnosis was not significant.	Nil

6

**Ha 2018**(51)<br><br>USA	Stage I-IIIA NSCLC treated with curative intent<br><br>n=177	Single centre, observational cohort study<br><br>2010 - 2017	Tumour board meeting to treatment initiation	PFS, OS	Non-significant	HR 1.0 (p=0.56) for overall survival in stage I-IIIA<br><br>HR 1.0 (p=0.74) for DFS in stage I only	Stage (localised)
**Huang 2020**(59)<br><br>Taiwan	Clinical stage I adenocarcinoma undergoing surgery<br><br>n=561	Single centre, observational cohort study<br><br>2006 – 2016	Radiological diagnosis to surgery (RDS)	OS	Non-significant	No significant difference in 5 year survival between timely vs delayed RDS	Stage (localised), surgery
			Histological diagnosis to surgery (HDS)		Timeliness advantageous	Timely HDS associated with improved 5 year survival, with HR 2.031 in multivariable model	
**Kanarek 2014**(55)<br><br>USA	Stage I-II NSCLC, undergoing resection<br><br>n=174	Single centre, observational cohort study<br><br>2003 - 2009	Diagnosis to surgical review, surgical review to treatment, diagnosis to treatment	Survival	Timeliness advantageous	Each week of delay from diagnosis to surgery increases HR by 1.04, adjusting for age, stage (IIB) and tumour size.	Stage (localised), surgery
**Kasymjanova 2017**(50)<br><br>Canada	All NSCLC receiving active treatment, inc targeted therapies<br><br>n=593	Single centre, observational cohort study<br><br>2010 - 2015	PC referral to first review; diagnosis to treatment; PC referral to treatment. Others treatment specific.	Survival	Timeliness advantageous	Delays >30 days from diagnosis to treatment associated with worse median survival (11 vs 14.8 months, p=0.04).	Stage (localised, regional, advanced)
**Khorana 2019**(40)<br><br>USA	All stage 1-2 NSCLC, excluding those without treatment or with delay >180 days<br><br>n=363,863	Registry (NCDB)<br><br>2004 - 2013	Diagnosis to treatment	OS	Timeliness advantageous	Longer time to treatment associated with worse OS in stage 1 and 2 disease undergoing surgery	Stage (localised), surgery
**Murai 2012**(47)<br><br>Japan	Stage 1 NSCLC undergoing SABR<br><br>n=201	Multicentre prospective cohort study (sub-analysis)<br><br>2004-2010	Diagnostic CT to treatment planning CT	Upstaging	Timeliness advantageous	Delays >4 weeks from diagnosis to planning CT are associated with increased upstaging (21% vs 0%).	Histology, stage (localised),
**Nadpara 2015**(33)<br><br>USA	All LC diagnoses age >66 years, from Medicare claims and SEER registry<br><br>n=42,089	Registry (SEER-Medicare)<br><br>2002 - 2007	CXR to first review; PC referral to first review; diagnosis to treatment; PC referral to treatment	Survival	Timeliness deleterious	Median survival 281 (271-291) vs 500 (479 - 520) days for timely vs delayed care. Overall survival reported as NSCLC vs SCLC, but not broken down by stage	Demographics, stage (localised, regional, advanced)

**Nadpara 2016**(34)<br><br>USA	Medicare beneficiaries aged >66 diagnosed with LC, care stratified as per clinical guidelines<br><br>n=1641	Registry (West Virginia Cancer Registry-Medicare)<br><br>2003-2006	CXR to first review; PC referral to first review; diagnosis to treatment; PC referral to treatment	Survival	Timeliness deleterious	Overall median survival no different in those receiving timely vs delayed care (299 vs 467 days, p=0.3), similar when stratified by stage and histology. However adjusted lung cancer mortality lower amongst patients receiving delayed care (HR 0.75, p<0.05), but full data not given.	Demographics
**Napolitano 2020**(37)<br><br>USA	Histologically confirmed NSCLC referred for surgery<br><br>n = 112	Single centre, observational cohort study<br><br>2013 – 2016	Time from first detection on CT to surgical resection	Upstaging	Non-significant	No significant difference between risk of upstaging in private vs Medicare insured (p=0.3), despite longer wait times for Medicare insured cohort	Demographics
**Navani 2015**(57)<br><br>UK	All radiological stage I-IIIA lung cancers, randomised to EBUS vs usual care for first diagnostic test<br><br>n=96	Multicentre RCT<br><br>2008 - 2011	First review to treatment decision	Survival	Timeliness advantageous	EBUS group experienced shorter time to treatment plan and improved median survival	Stage (localised), surgery
**Radzikowska 2012**(44)<br><br>Poland	Histologically confirmed NSCLC, any treatment modality<br><br>n=6384	Registry (Register of the National Tuberculosis and Lung Diseases Research Institute)<br><br>1995-1998	PC referral to first review; first review to first procedure; first review to diagnosis; diagnosis to treatment	OS	Timeliness deleterious	Secondary care delays <52 days associated with worse overall survival (HR 1.18, p=0.001)	Clinical factors
**Redaniel 2015**(42)<br><br>UK	All lung cancer diagnoses, defined by presence or absence of NICE 'alert' symptoms<br><br>n=5737*	Registry (Clinical Practice Research Datalink; Merged Cancer Registry; HES; ONS)<br><br>1998-2009	PC presentation to diagnosis	Survival	Mixed	Worse survival with intervals from first presentation to diagnosis of <1 month versus >6 months for patients without 'alert' symptoms, but no significant association in patients where 'alert' symptoms were present	Clinical factors
**Robinson 2015**(61)<br><br>Canada	All biopsy confirmed stage 3 NSCLC<br><br>n=237	Single centre, observational cohort study<br><br>2008 - 2012	Abnormal CT to oncology consultation; respiratory consultation to oncology consultation	Change in treatment intent	Non-significant	Patients who experienced weight loss or decline in performance status which resulted in a palliative approach to treatment did not have delayed care	Stage (regional)

**Samson 2015**(31)<br><br>USA	All clinical stage 1 NSCLC undergoing surgery<br><br>n=27,022	Single centre, observational case:control study plus registry (NCDB)<br><br>1998 - 2010	Diagnosis to treatment	Pathological upstaging, survival	Timeliness advantageous	Delays of ≥8 weeks from diagnosis to surgery associated with higher risk of pathological upstaging and reduced median survival.	Stage (localised), surgery
**Selva 2014**(63)<br><br>Spain	All NSCLC diagnosed either via rapid access referral route or (retrospective) via standard pathway<br><br>n=362	Single centre, 'quasi-interventional' case:control study<br><br>2005 - 2009	First secondary care appt booked to first treatment<br><br>Diagnosis to treatment interval	Upstaging	Non-significant	Rapid access reduced time to treatment but did not achieve a stage shift.	Intervention
**Shin 2013**(38)<br><br>South Korea	Histologically confirmed LC undergoing primary surgery<br><br>n=398	Registry (Korean Central Cancer Registry)<br><br>2006 - 2011	Diagnosis to treatment	OS	Non-significant	No association between time to surgery (<1 to >12 weeks) and all-cause mortality	Stage (localised), surgery
**Tsai 2020**(53)<br><br>Taiwan	Histologically confirmed NSCLC receiving active treatment<br><br>n=42,962	Registry (Taiwan Cancer Registry Database)<br><br>2004 – 2010	Histological diagnosis to treatment	OS	Mixed	Delays ≥7 days associated with increased relative risk of death in stage 1 (HR 1.45-2.41) and stage II disease (HR 1.21 – 1.58), but only significant for delays of >60 days in stage III, and non-significant for stage IV.	Stage (localised, regional, advanced)
**Vinod 2017**(48)<br><br>Australia	All NSCLC (any treatment)<br><br>n=1729	Registry (South Western Sydney Local Health Central Cancer Registry)<br><br>2006 - 2012	Diagnosis to treatment	Survival	Mixed	In patients with stage 3-4 NSCLC only, or stage 1-2 referred for palliative care, there was a marginal trend towards better survival in those who waited longer for treatment (mortality HR 0.99, p<0.05)	Stage (localised, regional, advanced), surgery, palliative
**Wai 2012**(60)<br><br>Canada	Unresectable stage 3 NSCLC<br><br>n=357	Case:control (2:1 radical vs palliative treatment intent)<br><br>1990-2000	First abnormal test to diagnosis; diagnosis to oncology referral; oncology review to treatment	Treatment intent	Non-significant	No significant difference between time to oncologist assessment and treatment intent.	Stage (regional)
**Wang 2012**(49)<br><br>USA	Inoperable stage 1-3 NSCLC with serial pre-treatment PET/CT scans<br><br>n=34	Multi-centre observational cohort study<br><br>2003 - 2010	First CT/PET to first treatment	Upstaging, PFS, OS	Timeliness advantageous	Inter-scan interval > 58 days associated with higher rates of progression (46.2% vs 4.8%, p=0.007).<br><br>Tumour growth rates and TTT were not associated with OS or PFS.	Stage (localised)

**Yang 2017**(58)<br><br>USA	Stage 1A squamous cell carcinoma undergoing surgery<br><br>n=4984	Registry (NCDB)<br><br>2006 - 2011	Diagnosis to treatment	Survival	Timeliness advantageous	Worse 5-year survival in those waiting >38 days from diagnosis to treatment	Stage (localised), surgery
**Yun 2012**(54)<br><br>South Korea	All lung cancer patients undergoing curative surgery<br><br>n=9097*	Registry (Korean Central Cancer Registry)<br><br>2001 - 2005	Diagnosis to treatment	Survival	Timeliness advantageous	Treatment delay >1 month associated with worse survival, particularly in low/medium volume centres	Stage (localised), surgery
**Živković 2014**(28)<br><br>Montenegro	All lung cancers diagnosed via single centre with >12 months follow up data available<br><br>n=151	Single centre, observational cohort study<br><br>2009	PC referral to first review; first review to diagnosis	Upstaging, survival	Non-significant	No association between time from referral to treatment and disease stage or survival.	Nil

(*) denotes total study sample size, where NSCLC forms an unspecified subgroup

CT = computed tomography; CWT: Cancer Waiting Times; EBUS = endobronchial ultrasound; HES = Hospital Episode Statistics; HR; hazard ratio; ISI = interscan interval; LC: lung cancer; MDT; multidisciplinary team; NCDB = National Cancer Database; NCRAS = National Cancer Registration and Analysis Service; NOS = not otherwise specified; NSCLC = non-small cell lung cancer; ONS = Office for National Statistics; OS = overall survival; PC = primary care; PET = positron emission tomography; PFS = progression free survival; RCT: randomised controlled trial; TTT: Time to treatment; UK: United Kingdom; US = United States of America

Figure 2: Reported median time intervals for included studies

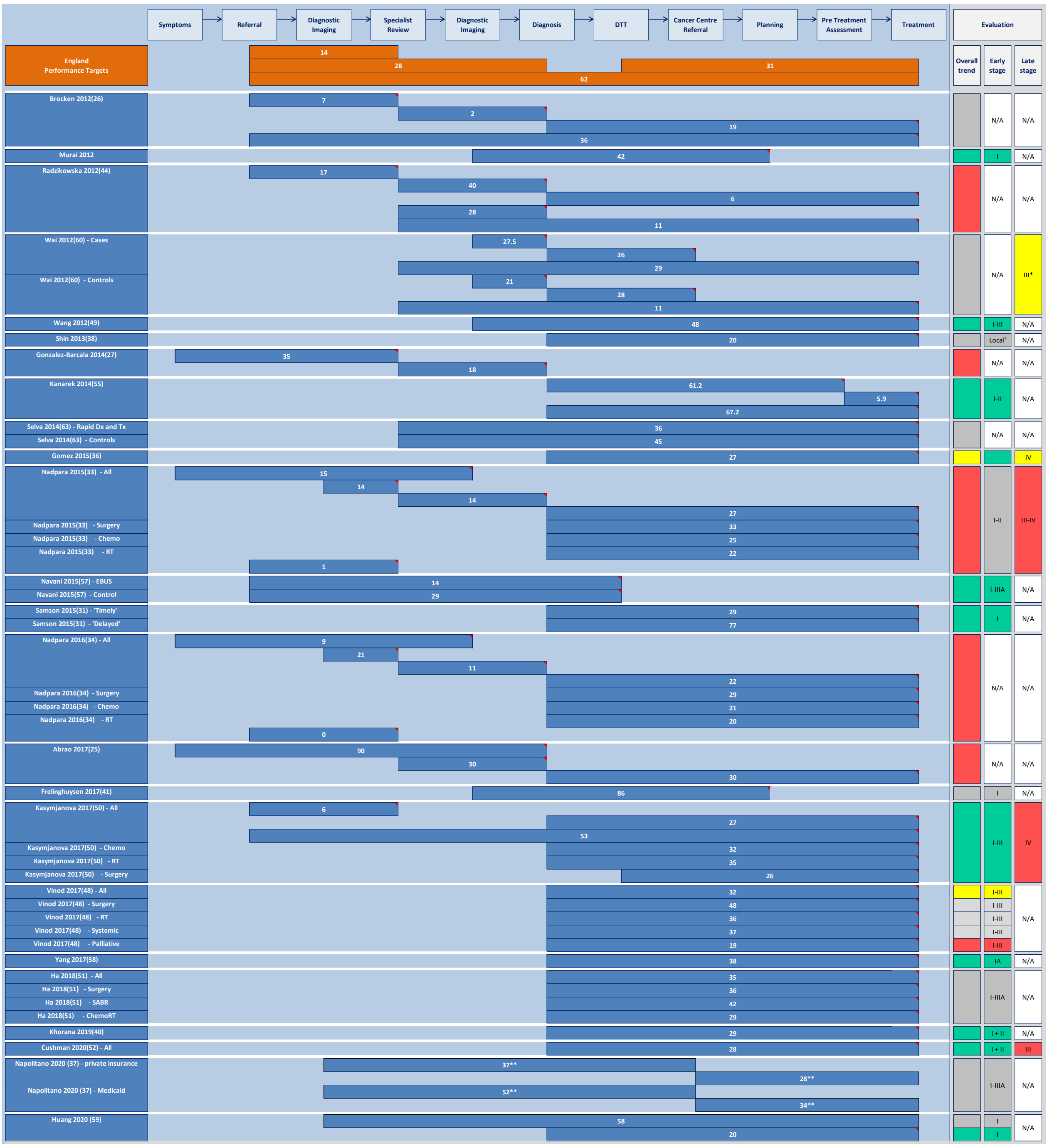

- Timeliness advantageous
- Mixed
- Timeliness deleterious
- Non-significant
- Not applicable

Chemo = chemotherapy; DTT = Decision To Treat; Dx = diagnosis; EBUS = endobronchial ultrasound, NOLCP = National Optimal Lung Cancer Pathway; RT = Radiotherapy; SABR = Stereotactive ablative radiotherapy; Tx = Treatment

*Wai 2012: Mixed associations observed for different time intervals between case and control groups
** Values reported as mean

11

Table 3a: Summary of evidence in early disease (*excludes studies only reporting surgical data, see Table 3d*)

	Study	Study design	Stage	Treatment	n	Time interval	Delay definition	Outcome measure	Trend	Outcome
**ALL TREATMENT MODALITIES**										
**STAGE I only**	Murai 2012(47)	Observational cohort (multi-centre)	I	Referred for SABR	201	Diagnostic CT to SABR planning CT	Interscan interval >4 weeks	Upstaging	Timeliness advantageous	Risk of upstaging 20.8% vs 0% (p=0.003) for delayed vs timely care.
	Nadpara 2015(33)	Observational cohort (registry)	I	Surgery, radiotherapy or chemotherapy	3,478	Diagnosis to treatment	>8 weeks from diagnosis to surgery<br>>7 weeks from diagnosis to chemotherapy<br>>6 weeks from diagnosis to radiotherapy	Lung cancer specific mortality	Non-significant	3yr survival rate 0.62 (0.6 - 0.64) vs 0.58 (0.55 - 0.62) for timely vs delayed
	Bullard 2017(39)	Observational cohort (registry)	‘Localised'	Surgery, chemotherapy or radiotherapy	185	Diagnosis to treatment	>42 days	Median survival	Non-significant	HR for mortality 0.98 (p=0.94) for timely vs delayed
	Frelinghuysen 2017(41)	Observational cohort	I	Referred for SABR	117	Diagnostic CT to SABR planning CT	NA	Upstaging, survival	Non-significant	Median ISI no different between stable T1, upstaged T1 and stable T2 lesions (p=0.4)
	Abrao 2018(46)	Observational cohort (single centre)	I	Any	30	Diagnosis to treatment	> 8 weeks	All-cause mortality	Non-significant	HR 1.24 (0.39-3.98, p=0.71) for delayed vs timely treatment
	Di Girolamo 2018(32)	Observational cohort (registry)	I	Any	6,158	GP referral to first review	>14 days	1 year net survival	Non-significant	88.8% (CI 87.9-89.7) vs 84.8% (78.7 - 91.0)
					15,363	Diagnosis to treatment	>31 days		Timeliness deleterious	89.3% (88.7 - 89.9) vs 95.6% (94.0 - 97.3)
					5,932	GP referral to treatment	>62 days		Non-significant	91.2% (90.1-92.3) vs 93.4% (92.1-94.6)
	Khorana 2019(40)	Observational cohort (registry)	I	Any	280,175	Diagnosis to treatment	>6 weeks	Overall survival	Timeliness advantageous	HR 1.032 (1.031-1.034, p<0.001) for each week delay

	Cushman 2020(52)	Observational cohort (registry)	I	Surgery, chemotherapy or radiotherapy	95,378	Histological diagnosis to treatment	>45 days	Overall survival	Timeliness advantageous	HR 1.15 (HR 1.12 – 1.17) for delayed vs timely
	Tsai 2020(53)	Observational cohort (registry)	I	Surgery, chemotherapy or radiotherapy	5,681	Histological diagnosis to treatment	Categorical (≤7 days, 8-14, 15-60, ≥61 days)	Overall survival	Timeliness advantageous	HR 1.45-2.41 for all intervals versus ≤7 days (p<0.001 for all)
**STAGE II only**	Nadpara 2015(33)	Observational cohort (registry)	II	Surgery, radiotherapy or chemotherapy	766	Diagnosis to treatment	>8 weeks from diagnosis to surgery<br>>7 weeks from diagnosis to chemotherapy<br>>6 weeks from diagnosis to radiotherapy	Lung cancer specific mortality	Non-significant	3yr survival rate 0.40 (0.36 - 0.45) vs 0.37 (0.30 - 0.44) for timely vs delayed
	Abrao 2018(46)	Observational cohort (single centre)	II	Any	26	Diagnosis to treatment	> 8 weeks	All-cause mortality	Timeliness advantageous	HR 3.08 (1.05 – 9.0, p=0.04) for delayed vs timely
	Di Girolamo 2018(32)	Observational cohort (registry)	II	Any	4,460	GP referral to first review	>14 days	1 year net survival	Non-significant	73.5% (72.1-74.9) vs 76.4% (68.0-84.7) for timely vs delayed
					8,614	Diagnosis to treatment	>31 days		Timeliness deleterious	74.4% (73.4-75.4) vs 86.1% (82.1-90.0) for timely vs delayed
					4,200	GP referral to treatment	>62 days		Timeliness deleterious	76.4% (74.6-78.2) vs 81.0% (78.9-83.0) for timely vs delayed
	Khorana 2019(40)	Observational cohort (registry)	II	Any	83,688	Diagnosis to treatment	>6 weeks	Overall survival	Timeliness advantageous	HR 1.016 (1.014 - 1.018, p<0.001) for each week delay for delayed vs timely
	Cushman 2020(52)	Observational cohort (registry)	II	Surgery, chemotherapy or radiotherapy	22,072	Histological diagnosis to treatment	>45 days	Overall survival	Timeliness advantageous	HR 1.05 (1.01 – 1.09) for delayed vs timely
	Tsai 2020(53)	Observational cohort (registry)	II	Surgery, chemotherapy or radiotherapy	1,526	Histological diagnosis to treatment	Categorical (≤7 days, 8-14, 15-60, ≥61 days)	Overall survival	Timeliness advantageous	HR 1.21-1.58 for all groups versus ≤7 days (p<0.05 for all)

**STAGE I-IIIA NOS**	Wang 2012(49)	Observational cohort (multi-centre)	I-III	Radiotherapy +/- concurrent chemotherapy	34	Diagnostic PET to treatment planning PET	ISI >58 days	Disease progression and upstaging	Timeliness advantageous	OR for disease progression 1.027 (p = 0.02) in delayed vs timely.
	Gomez 2015(36)	Observational cohort (registry)	'Localised'	Any surgery, radio- or chemotherapy, or combination	7,960	Diagnosis to treatment	> 35 days	All-cause mortality	Timeliness advantageous	HR 0.86 (0.8-0.91, p < 0.01) for timely vs delayed
	Navani 2015(57)	Multi-centre RCT: EBUS vs usual care as first diagnostic test	I-IIIA	All	96	First secondary care review to treatment decision	Intervention (median 15 days) vs control (median 30 days)	Survival	Timeliness advantageous	Median survival 503 days vs 312 days (p=0.038) in intervention vs control
	Kasymjanova 2017(50)	Observational cohort (single centre)	I-IIB	Any active treatment	177	Diagnosis to treatment	>30 days	Survival	Timeliness advantageous	HR for survival 2.07 (1.45-2.97, p<0.001) for timely vs delayed
	Vinod 2017(48)	Observational cohort (registry)	I-II	Any	375	Diagnosis to treatment	NS	Survival	Non-significant	All: HR 1 (1 - 1.01, p=0.25)
			I-III	Radiotherapy	288				Non-significant	Radiotherapy: HR 0.99 (p=0.11)
				Palliation	148				Timeliness deleterious	Palliative: HR 0.99 (0.98-0.99, p=0.02) for timely vs delayed
	Ha 2018(51)	Observational cohort (single centre)	I-IIIA	Surgery, radiotherapy, chemotherapy, combination or none	177	Tumour board meeting to treatment initiation	Guideline concordance	Overall survival	Non-significant	HR 1.0 (p=0.56) for survival
			I		122			Disease-free survival		Disease free survival in stage 1 subgroup (HR 1.0, p=0.74)

CT = computed tomography; GP = general practitioner (primary care); HR = hazard ratio; ISI = interscan interval; PET = positron emission tomography; SABR = stereotactic ablative radiotherapy

Table 3b: Summary of evidence in regional disease

Study	Study design	Stage	Treatment	n	Time interval	Delay definition	Outcome measure	Trend	Outcome
Wai 2012(60)	Case control (registry)	III	Chemoradiotherapy	119	Diagnosis to cancer centre referral	NA	Treatment intent	Timeliness advantageous	Median duration 26 days vs 28 days for radical CRT recipients vs palliative Tx, p=0.035
					Diagnosis to oncology consult			Non-significant	Median duration 31 days vs 31.5 days for radical CRT recipients vs palliative Tx, p=0.264
			Palliative	238	Oncologist review to start of treatment			Timeliness deleterious	Median duration 29 days vs 11 days for radical CRT recipients vs palliative, p <0.0001
Gomez 2015(36)	Observational cohort (registry)	'Regional'	Any surgery, radio- or chemotherapy, or combination	8,962	Diagnosis to treatment	> 35 days	All-cause mortality	Non-significant	HR 1.05 (0.8 - 0.91, p=0.054) for timely vs delayed treatment
Robinson 2015(61)	Observational cohort (single centre)	III	Radical vs palliative (any)	237	CT imaging to oncology consultation	NA	Treatment intent	Non-significant	No association between median time intervals and clinical deterioration impacting treatment intent
					Respiratory review to oncology review				
Nadpara 2015(33)	Observational cohort (registry)	III	Surgery, radiotherapy or chemotherapy	5,291	Diagnosis to treatment	>8 weeks from diagnosis to surgery	Lung cancer specific mortality	Timeliness deleterious	Median survival 305 days (*291 - 317) vs 472 days (443 - 498) for timely vs delayed treatment = * = 95% CI
						>7 weeks from diagnosis to chemotherapy			
						>6 weeks from diagnosis to radiotherapy			
Friedman 2016(62)	Observational cohort (single centre)	III	Any	109	First clinical review to treatment	NA	Overall survival	Non-significant	Patients seen by cancer board versus single clinician experienced faster treatment with borderline significant improved median survival (14 vs 17 months, p = 0.054)
Kasymjanova 2017(50)	Observational cohort (single centre)	III	Any active treatment	111	Diagnosis to treatment	>30 days	Overall survival	Timeliness advantageous	Median survival 17.2 vs 32.7 months for delayed vs timely treatment (p=0.04)

Hall H, *et al. Thorax* 2022; 77:762–768. doi: 10.1136/thoraxjnl-2021-216865

Bullard 2017(39)	Observational cohort (registry)	'Regional' II-III	Surgery, chemotherapy or radiotherapy	232	Diagnosis to treatment	>42 days	Survival	Non-significant	HR for mortality 1.18 (p=0.41) for timely vs delayed
Vinod 2017(48)	Observational cohort (registry)	III	Any	422	Diagnosis to treatment	NA	Survival	Timeliness deleterious	HR for mortality 0.99 (95% CI 0.99 – 0.99, p=0.03) for delayed vs timely
Abrao 2018(46)	Observational cohort (single centre)	III	Any	73	Diagnosis to treatment	> 8 weeks	All-cause mortality	Non-significant	HR 0.65 (0.38 - 1.1, p=0.11) for delayed vs timely treatment
Di Girolamo 2018(32)	Observational cohort (registry)	III	Any	14,453	GP referral to first review	>14 days	1 year net survival	Non-significant	48.1% (47.3-49.0) vs 46.2% (41.2-51.3)
Di Girolamo 2018(32)	Observational cohort (registry)	III	Any	23,667	Diagnosis to treatment	>31 days	1 year net survival	Timeliness deleterious	53.9% (53.3-54.6) vs 74.5% (69.7-79.2)
Di Girolamo 2018(32)	Observational cohort (registry)	III	Any	12,495	GP referral to treatment	>62 days	1 year net survival	Non-significant	52.4% (51.3-53.4) vs 65.2% (63.5-67.0)
Cushman 2020(52)	Observational cohort (registry)	III	Surgery, chemotherapy or radiotherapy	23,005	Histological diagnosis to treatment	>45 days	Overall survival	Timeliness deleterious	HR 0.93 (0.89-0.96) for delayed vs timely
Tsai 2020(53)	Observational cohort (registry)	III	Surgery, chemotherapy or radiotherapy	11,696	Histological diagnosis to treatment	Categorical (≤7 days, 8-14, 15-60, ≥61 days)	Overall survival	Timeliness advantageous	HR 1.13 for delays ≥61 days versus ≤7 days (p = 0.001)

CI = confidence interval; CRT = chemoradiotherapy; HR = hazard ratio; Tx = treatment

Hall H, *et al. Thorax* 2022; 77:762–768. doi: 10.1136/thoraxjnl-2021-216865

Table 3c: Summary of evidence in advanced disease

Study	Study design	Stage	Treatment	n	Time interval	Delay definition	Outcome measure	Trend	Outcome (timely vs delayed)
Nadpara 2015(33)	Observational cohort (registry)	IV	Surgery, radiotherapy or chemotherapy	7,212	Diagnosis to treatment	>8 weeks from diagnosis to surgery	Lung cancer specific mortality	Timeliness deleterious	Median survival 146 days (CI 140 - 152) vs 290 days (270-308) for timely vs delayed treatment
						>7 weeks from diagnosis to chemotherapy			
						>6 weeks from diagnosis to radiotherapy			
Gomez 2015(36)	Observational cohort (registry)	'Distant'	Surgery, radiotherapy or chemotherapy	11,810	Diagnosis to treatment	> 35 days	All-cause mortality (for those with survival <1 year vs >1 year)	Timeliness deleterious	HR 1.35 (1.28 - 1.42, p<0.001) for timely vs delayed treatment in patients surviving <1 year
								Timeliness advantageous	HR 0.86 (0.74-0.99, p=0.042) for timely vs delayed treatment in patients surviving ≥1 year
Kasymjanova 2017(50)	Observational cohort (single centre)	IV	Any active treatment	390	Diagnosis to treatment	>30 days	All-cause mortality	Timeliness deleterious	HR 0.72 (0.58-0.92, p = 0.008) for delayed vs timely treatment
Vinod 2017(48)	Observational cohort (registry)	IV	Any	878	Diagnosis to treatment	NS	Survival	Timeliness deleterious	HR for mortality 0.99 (95% CI 0.99 – 0.99, p=0.0008) for delayed vs timely
Bullard 2017(39)	Observational cohort (registry)	'Distant'	Surgery, radiotherapy or chemotherapy	329	Diagnosis to treatment	>6 weeks	Survival	Timeliness deleterious	HR for mortality 2.2 (p<0.001) for timely vs delayed
Abrao 2018(46)	Observational cohort (single centre)	IV	Any	230	Diagnosis to treatment	>8 weeks	All-cause mortality	Timeliness deleterious	HR for mortality 0.48 (0.35-0.66, p<0.001) for delayed vs timely
Di Girolamo 2018(32)	Observational cohort (registry)	IV	Any	22,460	GP referral to first review	>14 days	1 year net survival	Non-significant	23.3% (22.8 - 23.9) vs 19.5% (16.1-22.9)
				31,442	Diagnosis to treatment	>31 days		Timeliness deleterious	33.8% (33.2-34.3) vs 52.6% (45.0-60.2)
				14,665	GP referral to treatment	>62 days		Timeliness deleterious	33.8% (33.0-34.7) vs 44.6% (42.6-46.7)
Tsai 2020(53)	Observational cohort (registry)	IV	Surgery, chemotherapy or radiotherapy	24,059	Histological diagnosis to treatment	Categorical (≤7, 8-14, 15-60, ≥61 days)	Overall survival	Non-significant	No significant association between any delay and survival

GP = general practitioner; HR = hazard ratio

Table 3d: Summary of evidence in surgical cohorts

	Study	Study design	Stage	n	Time interval	Delay definition	Outcome measure	Trend	Outcome
**SURGERY ONLY**									
STAGE I only	Bott 2015(56)	Observational cohort (registry)	I	55,653	Diagnosis to treatment	>8 weeks	Pathological upstaging	Timeliness advantageous	HR 1.1 for upstaging (p=0.002) for delayed vs timely treatment
STAGE I only	Coughlin 2015(45)	Observational cohort (single centre)	I	180	Treatment decision to surgery	Categorical (months)	Upstaging	Non-significant	OR 0.216 (p=0.07) for delays of ≥3 months vs <1 month
							Survival		HR 1.064 (p=0.92) for delays of ≥3 months vs <1 month
STAGE I only	Samson 2015(31)	Case:control (registry)	I	13,511 'delayed'	Diagnosis to treatment	> 8 weeks	Survival, upstaging	Timeliness advantageous	Upstaging from clinical T1 significantly more likely in delayed vs timely (p=0.002)
				13,511 'timely'					Median survival 69.9 (+/- 1.3) months vs 57.7 (+/- 1.0) months for timely vs delayed, HR 1.004 per week delay
STAGE I only	Samson 2015(31)	Case:control (single centre)	I	449 'delayed'	Diagnosis to treatment	> 8 weeks	Upstaging	Timeliness deleterious	25% vs 16% for timely vs delayed (p=0.001)
				522 'timely'			Survival	Non-significant	Median survival 97.5 months (0.2-168.6) vs 90.5 (0-172.8)
STAGE I only	Yang 2017(58)	Observational cohort (registry)	IA	4,984	Diagnosis to treatment	>38 days	5 year survival	Timeliness advantageous	HR for death at 5 years 1.13 (1.02 – 1.25) in delayed vs timely care
STAGE I only	Khorana 2019(40)	Observational cohort (registry)	I	193,058	Diagnosis to treatment	>6 weeks	OS	Timeliness advantageous	HR 1.024 (1.022-1.026, p<0.001) for each week delay
STAGE I only	Huang 2020(59)	Observational cohort (single centre)	I	561	Radiological diagnosis to surgery (RDS)	>60 days	OS	Non-significant	5 year survival 83.3% vs 83.7% for timely vs delayed RDS (p = 0.57)
					Histological diagnosis to surgery (HDS)	>21 days		Timeliness advantageous	5 year survival 85.5% vs 75.9% for timely vs delayed HDS (p = 0.003). HR 2.031 in multivariate analysis.
STAGE II only	Coughlin 2015(45)	Observational cohort (single centre)	II	42	Treatment decision to surgery	Categorical (months)	Upstaging	Timeliness advantageous	OR 2.0 (p=0.02) for delays of ≥2 months vs <1 month
							Survival		HR 3.6 (p=0.036) for delays of ≥2 months vs <1 month

	Khorana 2019(40)	Observational cohort (registry)	II	49,386	Diagnosis to treatment	>6 weeks	OS	Timeliness advantageous	HR 1.017 (1.014-1.021) for each week delay
STAGE I-IIIA/NOS	Yun 2012(54)	Observational cohort (registry)	NS	9,094	Diagnosis to treatment	>31 days	5-year survival	Timeliness advantageous	HR 1.16 (1.06 - 1.27) for survival in timely vs delayed
	Shin 2013(38)	Observational cohort (registry)	'Local'	191	Diagnosis to treatment	>12 weeks	All-cause mortality	Non-significant	HR 0.79 (CI 0.42 – 1.48) for delays up to 12 weeks vs any shorter interval.
	Kanarek 2014(55)	Observational cohort (single centre)	I-IIA	174	Diagnosis to treatment	>42 days	Survival	Timeliness advantageous	HR 1.04 (CI 1.00 – 1.09) for each week's delay in surgery for stage I-II disease
	Navani 2015(57)	Multi-centre RCT	I-IIIA	29	First secondary care review to treatment decision	Intervention (median 15 days) vs control (median 30 days)	Survival	Non-significant	HR 0.37 (p=0.125) for survival in intervention vs control
	Vinod 2017(48)	Observational cohort (registry)	I-III	246	Diagnosis to treatment	NS	Survival	Non-significant	HR 1.01 (p=0.48) for timely vs delayed
	Cushman 2020(52)	Observational cohort (registry)	I-III	85,267	Histological diagnosis to treatment	>45 days	Overall survival	Timeliness advantageous	HR 1.14 (1.11 – 1.16) for delayed vs timely

HR = hazard ratio, NS = non-significant; OS = overall survival; RCT = randomised controlled trial

Table 4: Comparison of studies utilising National Cancer Database (NCDB)

Study	Years	Inclusion criteria	Exclusion criteria	Primary outcome measure
Bott 2015(56)	1998 – 2010	Clinical stage I NSCLC undergoing resection	Patients with T2b disease	Pathological upstaging
Samson 2015(31)	1998 – 2010	Clinical stage I NSCLC matched case:control for delayed vs timely surgery	Nil specified	Overall survival
Khorana 2019(40)	2004 – 2013	Stage I-II NSCLC (alongside other cancers)	No treatment received; first treatment >180 days from diagnosis; unable to establish treatment intervals; uncommon histology	Overall survival
Cushman 2020(52)	2004 – 2015	Non-metastatic NSCLC, treated with curative intent	Metastatic or unidentified stage' palliative treatment only; chemotherapy or immunotherapy alone; no treatment received; unknown treatment interval; first treatment >365 days from diagnosis	Overall survival
Yang 2020(58)	2006 - 2011	Clinical stage IA squamous cell carcinoma, undergoing lobectomy	Adjuvant chemo/radiotherapy; patients having surgery the same day as diagnosis (latterly included in sensitivity analysis)	Overall survival

Table E8a: Assessment of bias (observational studies)

1a. Are eligibility criteria, sources and methods of participant selection and follow-up clearly described? **1b.** Is the study population likely to be representative of the target population?
2a. Are demographic and characteristic data provided and complete? **2b.** Are reasons for non-participation included?
3a. Are missing data measured and accounted for?
4a. Are definitions for both time-intervals and outcome measures defined *a priori*? **4b.** Are the definitions appropriately measurable?
5a. Are statistical methods described? **5b.** Are confounding factors controlled for? **5c.** Is there consideration of potential waiting-time paradox?

Reference	1a.	1b.	2a.	2b.	3a.	4a.	4b.	5a.	5b.	5c.
Abrao 2017 (25)	Yes	Yes	Yes	Yes	Yes	Yes	Some symptom based	Yes	Unclear which	In discussion
Abrao 2018 (46)	Yes	Excluded unresectable disease diagnosed at surgery	Yes	Yes	NA	Yes	Yes	Yes	Yes	In discussion
Bott 2015 (56)	Yes	Yes	Yes	Yes	Yes	Yes	Yes	Yes	Yes	NA
Brocken 2012 (26)	Yes	Excluded stage IV	Yes	Yes	Yes	Yes	Yes	Yes	Yes	Yes
Bullard 2017 (39)	Yes	Yes	Yes	Yes	Yes	Yes	Yes	Yes	Yes	In discussion
Coughlin 2015 (45)	Yes	Yes	Yes	NA	Some	Yes	Yes	Yes	Yes	NA
Cushman 2020 (52)	Yes	Yes	Yes	Yes	Yes	Yes	Yes	Yes	Yes	Yes
Di Girolamo 2018 (32)	Yes	Yes	Yes	Yes	Yes	Yes	Yes	Yes	Some	Yes
Forrest 2015 (35)	Yes	Yes	Yes	NA	Yes	Yes	Yes	Yes	Yes	Yes
Frelinghuysen 2017 (41)	Yes	Excludes treatment within 25 days	Yes	Yes	Yes	Yes	Yes	Yes	No	Yes
Friedman 2016 (62)	Yes	Yes	Yes	Yes	NA	Yes	Yes	Yes	No	No
Geiger 2014 (29)	Yes	Yes	Yes	Yes	NA	Yes	Yes	Yes	Yes	No
Gomez 2015 (36)	Yes	Excludes palliative care	Yes	Yes	Yes	Yes	Yes	Yes	Yes	Yes
Gonzalez-Barcala 2014 (27)	Yes	Yes	Yes	Yes	Yes	Yes	Some symptom based	Yes	Yes	In discussion
Ha 2018 (51)	Yes	Veterans	Yes	Yes	Yes	Yes	Yes	Yes	Yes	In discussion
Huang 2020 (59)	Yes	Yes	Yes	Yes	Yes	Yes	Yes	Yes	Yes	NA
Kanarek 2014 (55)	Yes	Yes	Yes	Yes	Yes	Yes	Yes	Yes	Yes	In discussion
Kasymjanova 2017 (50)	Yes	Yes	Yes	Yes	Yes	Yes	Yes	Yes	Yes	Yes
Khorana 2019 (40)	Yes	Some exclusions	Yes	Yes	Unclear	Yes	Yes	Yes	Yes	NA
Murai 2012 (47)	Yes	Yes	Yes	Yes	NA	Yes	Yes	Yes	Yes	No
Nadpara 2015 (33)	Yes	Unclear	Yes	Yes	Yes	Yes	Some symptom based	Yes	Yes	Yes
Nadpara 2016 (34)	Yes	Yes	Yes	Yes	NA	Yes	Some symptom based	Yes	Yes but not shown	In discussion
Napolitano 2020 (37)	Yes	Single surgeon only	Yes	No	No	Yes	Yes	Yes	Some	No
Radzikowska 2012 (44)	Yes	Yes	Yes	NA	NA	Yes	Yes	Yes	Yes	Yes
Redaniel 2015 (42)	Yes	Yes	Yes	Yes	Yes	Yes	Some symptom based	Yes	Yes	Yes
Robinson 2015 (61)	Yes	Yes	Yes	Yes	Yes	Yes	Yes	Yes	No	No
Samson 2015 (31)	Yes	Yes	Yes	No	No	No	Yes	Yes	Yes	NA
Selva 2014 (63)	Yes	Yes	Yes	Yes	NA	Yes	Yes	Yes	Yes	In discussion
Shin 2013 (38)	Yes	Yes	Yes	Yes	Yes	Yes	Yes	Yes	Yes	In discussion
Tsai 2020 (53)	Yes	Yes	Yes	Yes	Yes	Yes	Yes	Yes	Yes	Yes
Vinod 2017 (48)	Yes	Yes	Yes	Yes	Yes	Yes	Yes	Yes	Yes	In discussion
Wai 2012 (60)	Yes	Yes	Incomplete	Yes	Yes	Yes	Yes	Yes	Yes	No

Wang 2012 (49)	Yes	Some	Yes	Yes	NA	Yes	Yes	Yes	No	No
Yang 2017 (58)	Yes	Yes	Yes	Yes	Yes	Yes	Yes	Yes	Yes	NA
Yun 2012 (54)	Yes	Yes	Yes	Yes	Yes	Yes	Yes	Yes	Yes	NA
Živković 2014 (28)	Yes	Yes	Some	NA	NA	Yes	Some symptom based	Some	Histology	In discussion

Table E8b: Assessment of bias (randomised controlled trials)

	**Selection bias**		**Performance bias**		**Detection bias**	**Attrition bias**	**Reporting bias**	**Other**
	**Random sequence generation**	**Allocation concealment**	**Blinding of participants**	**Blinding of personnel**	**Blinding of outcome assessment**	**Incomplete outcome data**	**Selective reporting**	**Other source of bias**
Navani 2015 (57)	Yes	Yes	Not possible	Not possible	Yes	No	No	No
